# Upregulation of Nuclear Factor-Related Kappa B Suggests a Disorder of Transcriptional Regulation in Minimal Change Nephrotic Syndrome

**DOI:** 10.1371/journal.pone.0030523

**Published:** 2012-01-23

**Authors:** Vincent Audard, André Pawlak, Marina Candelier, Philippe Lang, Djillali Sahali

**Affiliations:** 1 INSERM U 955, Créteil, France; 2 Université Paris-Est, Créteil, France; 3 AP-HP, Groupe Hospitalier Henri Mondor-Albert Chenevier, Service de Néphrologie, Créteil, France; 4 Institut Francilien de Recherche en Néphrologie et Transplantation, Henri Mondor Hospital, Creteil, France; Cardiovascular Research Institute Maastricht - Maastricht University, The Netherlands

## Abstract

Immune mechanisms underlying the pathophysiology of idiopathic nephrotic syndrome, the most frequent glomerular disease in children, are believed to involve a systemic disorder of T cell function and cell mediated immunity. How these perturbations take place remains unclear. We report here that NFRKB, a member of the chromatin remodeling complex, is upregulated in MCNS relapse, mainly in CD4+T cells and B cells and undergo post-translational modifications including sumoylation. We showed that NFRKB was highly expressed in nuclear compartment during the relapse, while it was restricted to cytoplasm in remission. NFRKB induced the activation of AP1 signaling pathway by upregulating the expression of c-jun. We showed that NFRKB promotes hypomethylation of genomic DNA, suggesting its implication in regulation of gene expression by enhancing the binding of transcription factors through chromatin remodeling. These results suggest for the first time that NFRKB may be involved in the disorders of transcriptional regulation commonly observed in MCNS relapse.

## Introduction

Idiopathic nephrotic syndrome (INS) is a kidney disease defined by a massive proteinuria and hypoalbuminaemia. Primary INS includes two major histological variants: minimal-change nephrotic syndrome (MCNS) and focal segmental glomerulosclerosis (FSGS), which account for 70% and 20% of INS in children, respectively and 25% each in adults [1], [2]. Both entities are considered as non-inflammatory diseases and are characterized by glomerular epithelial cell injury leading to massive proteinuria. Although MCNS and FSGS with relapse are considered as immunologically-mediated diseases, MCNS has rather a benign course, while the prognosis of FSGS may be more severe, depending of its sensitivity to steroid and immunosuppressive drugs.

MCNS is often triggered by immunogenic stimuli such as viral infections, immunizations or allergens [2]–[4]. Active disease is associated with alteration of both humoral immunity and cell-mediated immunity [4], [5]. The association of MCNS with primary immunological disorders such as Hodgkin's lymphoma [6], leukemia [7] and thymoma [8] support the hypothesis of a disorder of the immune system. Production of many cytokines is increased in relapses, suggesting that the disease is associated with perturbations of the transcriptional machinery [5], [9].

The chromosome DNA is tightly folded in complex with histone proteins, forming nucleosomes in a chromatin structure. Initiation of gene transcription is strongly inhibited on such a nucleosomal template [10]. Gene transcription requires restructuring of chromatin with nucleosomal unfolding, leading to a more open access to the DNA. The modifications of chromatin structure and properties include DNA methylation, histone modifications and functional miRNA processing. Compelling evidence supports the interdependence of these epigenetic mechanisms [11].

The INO80 chromatin remodeling complex is a large multisubunit ATP-dependent protein complex that regulates the assembly, disassembly of nucleosomes, facilitating their sliding along DNA. The function of INO80 complex involves transcriptional regulation, DNA repair and DNA replication [12]. The human INO80 complex includes several ATPases (Ino80p, Ruv1 Ruv2b), actin-related proteins (ARP) and non-conserved subunits including the Gli-Kruppel zinc finger transcription factor Yin-Yang 1 (YY1), the deubiquitylating enzyme Uch37 and nuclear factor related to kB (NFRKB), which has recently been identified as member of this complex [13]. Although the ATPases subunits mediate the ATP-dependent nucleosome remodeling activity, the precise function of the other subunits remains unclear. Evidence from microarray experiments revealed that the INO80 complex contributes to positive or negative regulation of transcription of up to 20% of genes in yeast [14], [15]. In human cells, the INO80 subunit YY1 recruits the complex and controls transcription of a large number of genes [16]. Currently, the role of NFRKB as chromatin remodeling factor remains unknown.

Molecular analysis of immune perturbations by subtractive cloning and differential screening from T cells of patients with MCNS led us to identify *NFRKB* among genes of which the expression is upregulated in relapse [17]. The *NFRKB* gene is localized in 11q24-q25 and encodes for 25 exons spanning 29,234 bp. The full-length mRNA contains 5046 bp, which encodes for a 1324 amino acid-protein with a predicted molecular weight of 150 kDa. Our knowledge about the function of *NFRKB* is very limited so that it is difficult to understand its potential implication in immune cell disturbances occurring during MCNS relapse. Thus, we carried out functional studies aiming to characterize the NFRKB-mediated signaling pathways.

## Results

### Isolation and characterization of NFRKB

We identified the NFRKB transcript by differential screening of a cDNA library using subtractive cDNA probes prepared from lymphocyte mRNAs isolated from the same patient during the relapse and remission phases [17]. The full-length transcript contains 5046 nucleotides with a coding sequence of 3972 nucleotides, while the 3′- and 5′-UTR consist of 201 bp and 950 bp, respectively.

**Figure 1:**
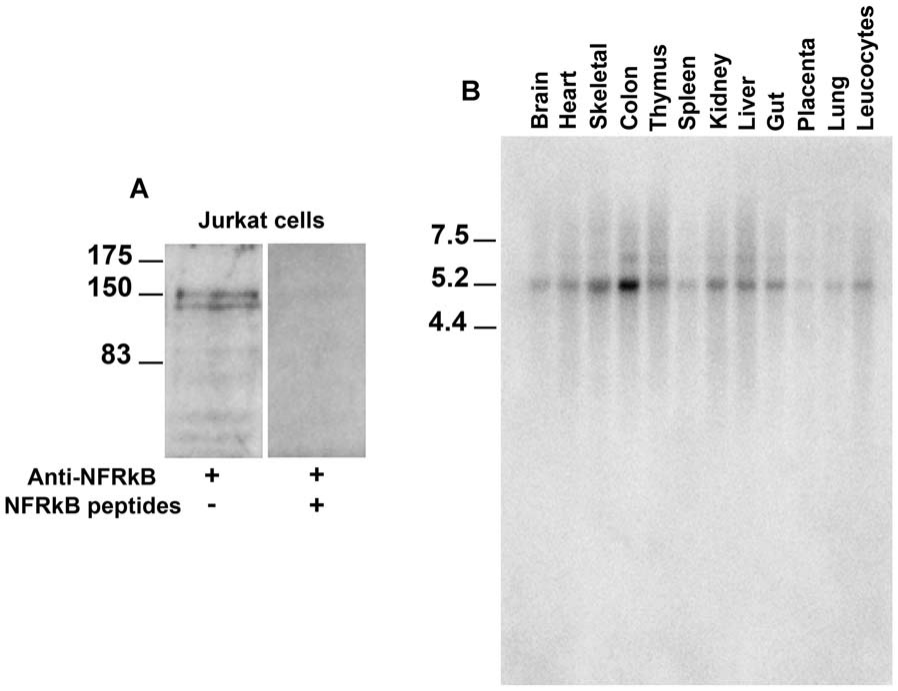
Expression of NFRKB in Jurkat cells. **A,** Western blot of total lysates using anti-NFRKB reveals a double band at the expected size. The specificity was attested by the loss of the binding upon preincubation of antibody with synthetic peptides corresponding to positions 543–557 and 1091–1106 within the primary structure of NFRKB. These data are representative of three independent experiments. **B,** **Northern blot analysis of NFRKB.** Multiple tissue-Northern blot (Clontech Laboratories, Inc.) was hybridized with a full-length-probe and exposed to Phosphoimager Storm for 24 h. This experiment was repeated once.

The predicted NFRKB protein contains 1324 amino acids. Its primary structure consists of several interacting docking sites including five PKC domains, an Erk domain, a GSK3 kinase domain, four Src homology 3 (SH3) domains and a bipartite nuclear signal (http://scansite.mit.edu/cgi-bin/motifscan). Immunoblotting of protein lysates from Jurkat cell with anti-NFRKB antibody detected a protein-doublet, corresponding to two isoforms (Access numbers P_001137307.1 and NP_006156.2) with an apparent molecular weight of 145–150 kDa (Fig. 1A), in close agreement with the molecular mass estimate deduced from amino acid content (142 kDa). The specificity of the labeling was checked by the loss of the signal following preincubation of the antibody with NFRKB peptides (Fig. 1A).

We used commercial Northern-blot (each line containing 2 microgr of polyA RNA) to explore the NFRKB expression in different tissues, using a radiolabelled cDNA corresponding to entire coding sequence of NFRKB. The transcript was basically detected at low levels, except in the colon that exhibited a higher expression (Fig. 1B).

### Expression of NFRKB in patients with steroid sensitive MCNS

**Figure 2:**
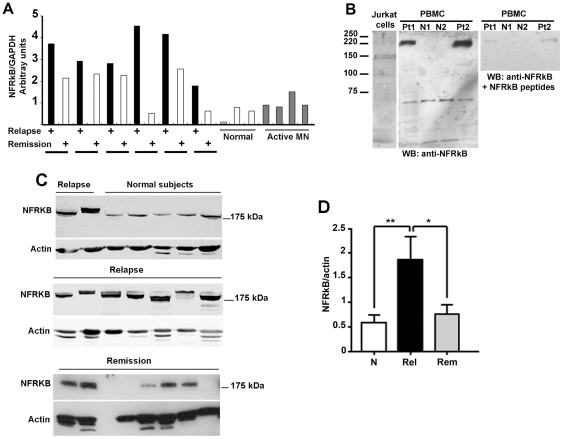
Differential expression of NFRKB in MCNS. **A**, RT-PCR analyses in six patients with MCNS during the relapse and remission phase. The top panel shows the quantification of PCR products, as determined using the Image Quant, version 1.11, analysis software, after normalization against the corresponding GAPDH mRNA values (▪, relapse; □, remission). These data are representative of two independent experiments. **B**, Western blots of PBMC protein lysates using anti-NFRKB antibody reveals a larger size of the protein (≈220 kDa), compared with the band visualized in jurkat cell lysates. The specificity was confirmed by the loss of the binding upon preincubation of antibody with specific peptides. These data are representative of three independent experiments. **C**, Western blot analysis of NFRKB abundance in PBMC of patients with MCNS relapse or remission and in normal subjects. These data are representative of three independent experiments. **D**, The relative abundance of NFRKB was assessed by quantifying the specific band after normalization with the corresponding actin, using the Image J software. The amount of NFRKB is significantly increased in relapse, as compared with remission or normal subjects (*p<0.05; ** p<0.01, unpaired t test).

We analyzed the expression of *NFRKB* transcript in peripheral blood mononuclear cells (PBMC) from six patients with MCNS in whom both relapse and remission (free of steroid therapy) samples were available. The transcript was increased in relapse, as compared with normal subjects (Fig. 2A). In remission, the *NFRKB* level was diminished in some patients but remained above the values observed in normal subjects. The expression of *NFRKB* in active membranous nephropathy (MN) was not significantly different from normal controls, which suggests that the upregulation of *NFRKB* is apparently not a consequence of nephrotic syndrome.

**Figure 3:**
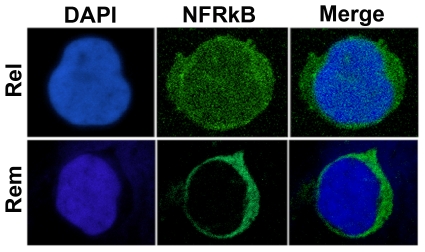
Confocal microscopy analysis of NFRKB staining in MCNS PBMC. Immunofluorescence of PBMC from relapse and remission with anti-NFRKB antibody. Note that NFRKB is expressed in nuclei and cytoplasm compartments during the relapse but it is restricted to cytoplasm in remission. These data are representative of three independent experiments.

Next, we studied the expression of NFRKB in PBMC protein lysates from patients with MCNS and normal subjects. Unexpectedly, anti-NFRKB antibody recognized a band with an apparent molecular weight of 220 kDa (Fig. 2B). This signal was abrogated following preincubation of antibody with the immunogenic peptides. We next screened several PBMC samples from patients with MCNS relapse (n = 13) or remission (n = 9) as well as in normal subjects (n = 12). The relative abundance of NFRKB was significantly increased in relapsed as compared with remission or normal subjects (Fig. 2C). In some patients, the abundance of both NFRKB isoforms were increased, while in some others, only one isoform was selectively increased. The reasons of this variability are unclear. These data are summarized in Fig. 2D. Confocal microscopy analysis was performed in PBMC from six patients with steroid sensitive MCNS, in relapse and remission phases. We observed that near than 50% of PBMC were positive. NFRKB was highly expressed in a diffuse fashion in nuclear as well as in the cytoplasm compartments during the relapse phase, while in remission, its expression was lower and restricted to cytoplasm compartment (Fig. 3).

**Figure 4:**
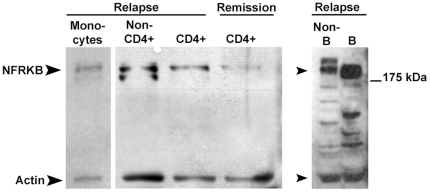
Expression of NFRKB in PBMC subsets. Western blots of protein lysates from PBMC subsets using anti-NFRKB antibody. Note that NFRKB is predominantly expressed in CD4+ cells and B cells in MCNS relapse. These data are representative of two independent experiments.

To determine whether NFRKB is expressed by a particular subpopulation of PBMC, we immunoblotted protein extracts from cell fractions isolated by immunoselection of PBMC from patients with relapse. The Fig. 4 shows that NFRKB was mostly upregulated in CD4+T cells and B cells from relapse, with a lesser amount produced in monocytes.

**Figure 5:**
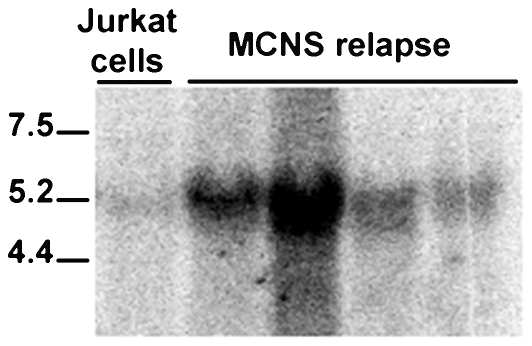
Northern blot analysis of NFRKB in MCNS. Northern blot of PBMC total RNA from four patients with MCNS relapse, was hybridized with a full-length-probe and exposed to Phosphoimager Storm for 24 h. This experiment was repeated once.

The discrepancy between the apparent molecular weight detected in Jurkat cells and in MCNS patients led us to investigate whether NFRKB was differentially spliced in the disease. We performed Northern-blot analysis using total RNA from patients with relapse. We only visualized a 5.2 kb-transcript, which co-migrates with the mature NFRKB mRNA detected in jurkat cells (Fig. 5). These results suggest that patients with MCNS did not display abnormal spliced NFRKB forms, but that the post-translational modifications likely account for the higher observed bands.

### Post-translational modifications of NFRKB include N-glycosylation and sumoylation

**Figure 6:**
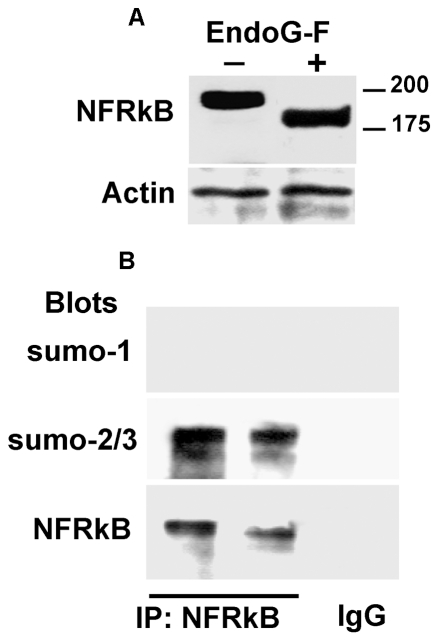
Post-translational modifications of NFRKB. **A**, representative experiment of N-glycosidase-F traitement. Total protein lysates were purified from PBMC, treated with the N-glycosidase-F, then immunoblotted with anti-NFRKB antibody. Note that the band size is reduced by ∼20 kDa. **B**, Immunoprecipitation of NFRKB from PBMC protein lysates followed by immunoblotting with anti-sumo-1 or anti-sumo-2/3. These data are representative of two independent experiments.

Because the asparagine residues are highly represented in the predicted structure of NFRKB, we studied the effects of endoglycosidase-F, an enzyme that cleaves the glycosidic bond between two N-acetylglucosamine residues. The Fig. 6 (upper panel) shows mobility electrophoretic of NFRKB incubated in the absence or in the presence of endoglycosidase-F. The NFRKB band decreased from 220 kDa to 200 kDa after endoglycosidase-F treatment, yet higher than the immunoreactive band detected in jurkat cells. In addition, four high probable putative sites for sumoylation were identified in NFRKB (VKEE2_11–215_, IKSE_351–354;_IKSE_382–386_ and VKYD_663–666_). To assess whether the sumoylation of NFRKB might account for the apparent higher molecular weight, we performed immunoprecipitation from PBMC protein lysates of two MCNS relapse samples and eluates were detected either with anti-sumo-1 or anti-sumo-2/3 antibodies and then reprobed with anti-NFRKB. As shown in Fig. 6 (lower panel), anti-sumo-2/3 reacted with a major 220 kDa-band, which was specifically recognized by anti-NFRKB antibody. A minor sumo-2/3 reactive band was also faintly recognized by anti-NFRKB. By contrast, we did not detect any signal with anti-sumo-1 antibody, suggesting that NFRKB did not contain sumo-1.

### NFRKB positively regulates AP1 signaling pathway by upregulating the expression of c-jun

**Figure 7:**
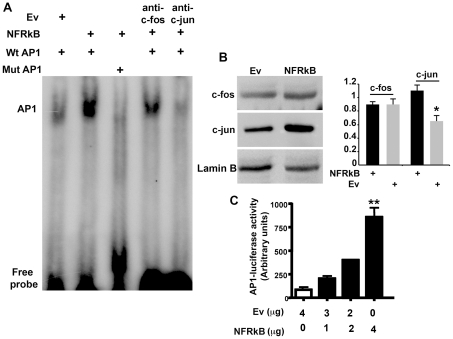
NFRKB induces activation of API transcripton factor. **A,** AP1 DNA-binding activity of 25 microg of nuclear proteins. Nuclear extracts from NFRKB-transfected HEK cells were incubated with the wild-type AP1 oligonucleotide (Wt AP1) or with mutant-type AP1(Mut AP1) oligonucleotide. **B,** Expression of c-fos and c-jun. Immunoblots were performed with 50 microg of proteins. **C,** HEK cells were transiently co-transfected with the NFRKB or empty vector (Ev), and AP1-Luc reporter plasmid. The luciferase activity was measured using the “Dual Luciferase reporter Assay”. The data are presented as relative luciferase activity (firefly luciferase/the renilla luciferase). Statistical analyses were carried out on data from five independent experiments using the one-way Anova (**p<0.015; Kruskal-Wallis test).

Because NFRKB was highly expressed in nuclear compartment during the relapse, we sought to determine whether it influences different signaling pathways. We studied by EMSA experiments the activation of NF-kB, ISRE, NFAT and AP1 in HEK cells transfected with NFRKB expression plasmid or empty vector. Overexpression of NFRKB increased AP1 DNA binding activity (Fig. 7A), while we did not found any effect on other transcription factor activities (data not shown). The specificity of the binding was attested by the loss of the shift when nuclear extracts were incubated with the mutated probe or in the presence of anti-cjun antibody. AP-1 activity is induced, among others, by cytokines and growth factors. In many cell types, including the immune cells, AP1 activity is composed primarily of Jun and Fos heterodimers [18]. The induction of AP1 binding activity was associated with an increased abundance of c-jun in nuclear extracts of HEK cells transfected with NFRKB, as shown in Fig. 7B. To examine whether binding to AP1 DNA binding site was functionnaly active, we performed luciferase assay in HEK cells. The AP1-luc reporter was transfected into HEK cells with a full-length NFRKB expression construct or the empty vector. Whereas the luciferase activity was very lower in the presence of the empty vector, NFRKB induces AP1 activity in a dose-dependent fashion (Fig. 7C).

### NFRKB induces hypomethylation of genomic DNA

**Figure 8:**
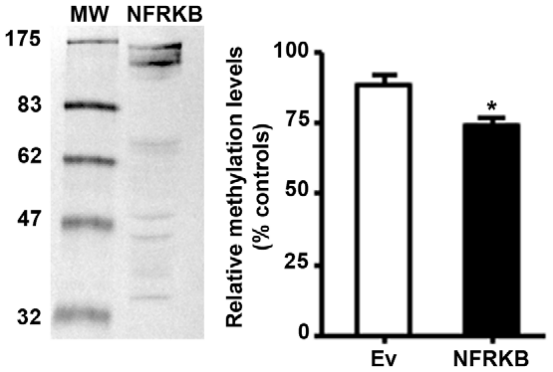
NFRKB induces hypomethylation of genomic DNA. Genomic DNA was prepared from NFRKB-transfected HEK cells or empty vector and methylation assay was performed as described in Materials and Methods. Statistical analyses were carried out on data from five independent experiments using the two-tailed *t*-test (*p<0.05; unpaired *t*-test).

The regulation of gene expression through epigenetic mechanisms such as DNA methylation and chromatin structure modification plays an important role in the regulation of gene expression. Because NFRKB belongs to INO80 complex, which is involved in chromatin remodeling activity, we examined whether NFRKB may influence DNA accessibility by monitoring genomic DNA methylation in HEK cells transfected with NFRKB expression plasmid. As shown in Fig. 8, the percentage of methylated DNA was decreased in NFRKB-overexpressing HEK cells (mean± SD: 74.14±3,64) relatively to cells transfected with empty vector (mean± SD: 88.25±3,74, *p<0.05). This result suggests that NFRKB promotes hypomethylation of genomic DNA, which may facilitate gene transcription.

## Discussion

MCNS is associated with immune disturbances of which the molecular mechanisms remain unclear [4], [5]. In this study, we show that NFRKB is recruited in active MCNS and display some unexpected features. We provide evidence that: i) the NFRKB abundance was increased in MCNS CD4+ T cells and B cells and undergo post*-*translational modifications including sumoylation; ii) NFRKB was highly expressed in nuclear compartment during the relapse, while it was restricted to cytoplasm in remission; iii) NFRKB induced the activation of AP1 signaling pathway by upregulating the expression of c-jun; iv) NFRKB increases hypomethylation of genomic DNA suggesting its involvement in the regulation of gene expression through chromatin remodeling, which enhances the binding of specific transcription factors.

Sumoylation of NFRKB is consistent with its function in transcriptional regulation. Sumoylation can either enhance or inhibit the transcription of target genes, although a majority of studies have identified a functional role of sumoylation in transcriptional repression [19]. Because NFRKB was highly sumoylated in MCNS relapse, while the NFRKB protein detected by Western blotting in HEK cells after transfection was primarily non sumoylated, we cannot conclude whether NFRKB enhances or represses transcription in vivo, notably in relapse. Sumoylation may also regulate the nucleo-cytoplasmic traffic of proteins [20]. NFRKB contains two sumoylation sites localized in the vicinity (IKSE_382–386_) or into the bipartite nuclear signal (IKSE_351–354_). The shuttling of NFRKB between nuclear and cytoplasm compartments during the MCNS relapse and remission phases could reflect differential sumoylation.

AP-1 activation occurs in response to a wide range of stimuli involved in cellular processes, which promote proliferation, differentiation, survival and apoptosis [21]. AP-1 protein function is regulated primarily by phosphorylation [21] and by redox potential [22]. In unstimulated T cells, AP-1 expression is low, but there is a rapid induction of AP-1 activity after T cell stimulation. The lack of AP-1 activation correlated with markedly decreased IL-2 synthesis and anergic T cells exhibit defective AP-1 activity [23]. AP-1 regulates at least in part a number of cytokines directly or through a cooperation with other transcription factors. Although both Th1 and Th2 express AP-1 after activation, higher levels of c-Fos and c-Jun are found in Th2 cells [24]. B cell activation, both T cell–dependent and T cell-independent can lead to AP-1 activation, through recruitment of PKC and/or CD40 pathways [25], [26].

The potential roles of epigenetic alterations in the pathogenesis of MCNS disease are suspected on the following arguments: 1) MCNS relapses are frequently triggered by an external or internal environmental factor including viral infection, toxin, nutriments, exposition to chemical products and stress [5], [27]. 2) A genetic defect cannot explain the relapsing-remitting profile of the disease but epigenetic alterations may occur without a direct change in the genetic sequence and are potentially reversible. 3) Epigenetic alterations have been reported in autoimmune disease with frequent/remission phases such as systemic lupus erythematosus and rheumatic diseases [28]. 4) Steroid therapy may induce remission by reversing epigenetic changes [29].

The lymphoid tissues associated with the intestine are exposed continuously to food antigen and are the largest part of the immune system. After feeding, T cells are activated locally, in the intestine-associated lymphoid organs, and in peripheral lymphoid tissues [30]. Each of these sites contains distinctive populations of antigen presenting cells and has unique local microenvironments that may influence the immune response. Inappropriate responses to food proteins might be responsible for immunopathological diseases such as food hypersensitivity, inflammatory bowel diseases and glomerular diseases. The association between celiac disease and MCNS has been reported [31] and could be more frequent in children from developing countries. However, the potential disturbance of mucosal immunity has little explored in MCNS.

Epigenetic mechanisms include DNA methylation, post-translational modifications of histone tails and changes in miRNA expression. The INO80 complex functions as an epigenetic regulator of gene expression via the modifications of chromatin structure. Chromatin remodeling complexes facilitate the access of factors that mediate transcription by modulating nucleosome position and/or composition. Inhibition of DNA methylation by some drugs, such as hydralazine and procainamide, can cause lupus-like disease characterized by excessive and inappropriate transcription. It is now believed that DNA demethylation alters T cell genes expression and contribute to lupus pathogenesis. A genetic basis for idiopathic MCNS has not been demontrasted. Because most patients (∼80%) are steroid sensitive, it is unlikely that a genetic defect, such as identified in steroid resistant nephrotic syndrome, may explain the pathogenesis of the disease. On the other hand, the upregulation of NFRKB in MCNS suggests that epigenetic mechanisms may be involved in immune disturbances. A wide variety of immunological abnormalities in MCNS, including observations in vivo and in vitro, affecting both humoral immunity and cell-mediated immunity has been reported [4]. The cytokine production in MCNS is complex and includes T helper 1 (interferon gamma) and T helper 2 (IL13) suggesting a disorder of transcriptional regulation [4]. A more salient finding in patients with MCNS is the presence of hypogammaglobulinemia, which cannot be explained by the urinary loss alone, because only some IgG fractions (IgG1 and IgG2) seem depressed [4]. The mechanism of hypogammaglobulinemia is unclear.

MCNS relapses often occur following a rapid contact with a variety of antigens present in the environment. How the environment modifies the immune system through epigenetic mechanisms to cause relapse, remains to be clarified.

## Materials and Methods

### Patients

The cohort of adult patients analyzed in this study is from our clinical department. All adult patients with MCNS relapse had proteinuria above 3 g/24 h and severe hypo-albuminemia at the time of blood sampling, which was performed before the beginning of steroid treatment. The diagnosis of kidney disease was confirmed by renal biopsy. MCNS and MN were clinically classified as idiopathic in all cases. All patients with MCNS investigated in this work were steroid sensitive. The demographic, clinical and biological characteristics of patients are summarized in Table 1.

**Table 1:** Demographic, clinical and biological characteristics of patients and normal subjects.

	Adults with MCNS^a^	Adults with MN^a^	Normal Adults
Number of patients	13	4	12
Age (years)^b^	25 (18–39)	30 (25–49)	28 (25–32)
Gender (male/female)	8/5	3/1	7/5
Proteinuria (g/day)	8 (6–25)	5 (3,5–8)	negative
Albuminemia (normal 39,5–49 g/L)	14 (8–23)	19 (14–28)	NT^d^
Plasma creatinine (micromol/l)	95 (75–135)	90 (80–120)	NT
Steroid therapy^c^	Relapse: noneRemission: none	none	none

a Diagnosis was established by renal biopsy.

b The data are presented as mean and extremes values are in parentheses.

c At the time of blood sampling.

d NT: not tested.

### Identification of NFRKB

The transcript NFRKB (Accession number: NM_001143835) has been isolated by subtractive and differential screening of a cDNA library constructed from peripheral T lymphocytes of patient with MCNS relapse [17].

### Northern Blot

Human multiple-tissue Northern blot (CLONTECH Laboratories, Inc.) was hybridized with a cDNA probe corresponding to the full-length coding sequence. Hybridization and blot processing were performed as described previously [17].

### Purification of PBMC and cell Subsets

Human PBMC were purified through a Ficoll/Hypaque density gradient (Eurobio, France). The subpopulations of T lymphocytes, B lymphocytes and monocytes were isolated by immunomagnetic selection, using a cocktail of hapten conjugated antibody specific of each corresponding subset (Miltenyi Biotech, Auburn, CA).

### Reverse transcription-polymerase chain reaction (RT-PCR)

Total RNA was prepared from the PBMC using an RNeasy kit (Qiagen, Chatsworth, CA, USA). One microg of RNA was reverse transcribed in the presence of Oligo(dT)_12–18_ primers, using a SuperScript™ First-Strand Synthesis System (Invitrogen, CA). Samples (2 microl of the RT reaction mixture, corresponding to 50 ng of total RNA) were amplified in a 25 microl reaction mixture containing 0.2 mM of each NFRKB primer (Table 2) and 0.5 microl of DyNAzyme™ EXT DNA polymerase (Finnzyme, Finland). PCR reaction was performed on a GeneAmp 2700 (Applied Biosystem, CA, USA), using the following programs: an initial denaturation step at 94°C for 3 min, following by 25 cycles (94°C for 15 sec, 60°C for 30 sec, and 68°C for 2 min), and by a final extension at 68°C for 10 min. The reaction product was normalized to the amount of GAPDH product amplified in parallel, using the appropriate primers (Table 2). The PCR products were separated on a 1.5% agarose gel, and the intensity of the specific bands was quantified using ImageJ 1.42 software (www.rsb.info.nih.gov).

**Table 2:** Sequence of primers and PCR conditions.

Primers	Sequence	Accessionnumber	Expectedsize	AnnTemp(°C)	PCRcycles
Human NFRKBFull length	ForwardGGGGACAAGTTTGTACAAAAAAGCAGGCTTCCAT GTCTTTGTCCAGAGTACCTGTGGAReverseGGGGACCACTTTGTACAAGAAAGCTGGGTC CTATTGTTGCTCAGGTGCCTGTTTAGGAGACG	NM_006165	3972	64	40
PCR primers	NFRKBForward: CTCTGGAACTTGGTCCGTGTGGAGATReverse: CAGGACTAGGAGAACGTGCTGGAGAGC		657	60	32
	GAPDHForward: ACCACAGTCCATGCCATCACReverse: TCCACCACCCTGTTGCTGTA	NM_004048	374	58	25

The underlined sequences correspond to attB-sites used to incorporate the PCR product into the gateway plasmids.

### Confocal microscopy analyses

Immunostaining of PBMC or cell subpopulations was performed as previously described [9].

### Plasmid constructs, cell culture, transient transfections and luciferase assays

Total RNA was isolated using a Rneasy kit (Qiagen, Chatsworth, CA). The NFRKB coding sequences was amplified by RT-PCR using the primers that are suitable for cDNA constructs in the gateway system (Invitrogen, Inc, CA). The NFRKB mRNA was prepared from patients with MCNS. Reverse transcription was performed with Superscript II (Invitrogen, Inc, CA) and PCR amplification with DyNazyme EXT DNA polymerase (Finnzyme, Finland) using the following conditions: an initial denaturation step at 94°C for 2 min, following by 35 cycles (94°C for 15 sec, 64°C for 30 sec, and 72°C for 5 min), and by a final extension at 72°C for 10 min. The cDNA product was inserted in the pDonor plasmid. The quality of cDNA was checked by sequencing. The full-length NFRKB was transferred into pDest40 by recombination, using the LR recombinase (Invitrogen, Inc, CA).

Jurkat cells were cultured at 0.5×10^6^ cells/ml in complete RPMI medium containing 10% fetal calf serum (FCS), 50 microg/ml Penicillin, 100 microg/ml Streptomycine and 2 mM Glutamine (Invitogen, France). Human embryonic kidney (HEK) cells were obtained from the American type Culture Collection. The cells were maintained in complete DMEM medium and were transiently transfected with one microg DNA per 10^6^ cells using the nanofectine method according the instructions provided by the manufacturer (PAA, Austria). The cells were allowed to recover for 24 hours, washed three times in cold PBS and processed for analysis. To minimize variations in transfection efficiency, the same cell passage number per cell line was used for all transfections. The AP-1-luc (Promega, France) vector contains the firefly luciferase gene under the control of AP-1 enhancer element. A phRL-null vector (Promega, France), containing a renilla luciferase gene was used as an internal control for transfection and luciferase assays.

### Immunoprecipitations and Western blot analyses

Primary antibodies used in this study included, anti-sumo-1 (sc-5308) anti-sumo-2/3 (sc-32873), anti-c-fos (sc-7202), antic-jun (sc-1694) anti-lamin B (sc-6216) (Santa Cruz, Biotechnology, Inc, CA) and anti-V5 (Invitrogen, Inc, CA). We generate a rabbit polyclonal antibody raised against two synthetic peptides corresponding to positions 543–557 and 1091–1106 within the predicted structure of NFRKB, respectively.

Cell protein extracts from cell lines (Jurkat or HEK 293) were prepared in lysis buffer containing 150 mM NaCl, 10 mM Tris HCl pH 7.5, 2 mM DTT, 10% glycerol, 1 mM EDTA, 1% NP40, 1 mM protease inhibitors, 1 mM Na fluoride and 1 mM sodium orthovanadate). For immunoprecipitation, cell lysates containing equal amount of protein were precleared with protein G-sepharose (GE Helthcare Bio-science AB, Upssala, Sweden) for 1 h at 4°C. The beads were presaturated with 5% BSA for 4 h before use. After preclearing, protein lysates were incubated with the appropriate antibody for 2 h at 4°C, then 50 microl of protein G-sepharose beads were added and the incubation was continued overnight at 4°C. The beads were washed six times with the lysis buffer B containing 0,1% NP40 and bound proteins were resolved by 10% SDS-PAGE, transferred on nitrocellulose membrane and processed for immunoblotting. For controls of immunoprecipitations, the non-immune rabbit or mouse IgG (Alpha Diagnostics Intl. Inc., San Antonio, USA) were used instead of primary antibody.

### Endoglycosidase treatment

Treatment of PBMC total lysates with recombinant N-glycosidase-F (0.4 unit/ml, Roche Diagnostic, France) was carried out at 22°C for 18 h in 100 mM-sodium phosphate, pH 6.0, containing 75 mM beta-mercaptoethanol, 50 mM-EDTA, 0.5% (w/v) Triton X-100, 0.05% (w/v) SDS. The enzymatic digestion was terminated by addition of equal volume of Laemmli buffer. The samples were then migrated in SDS/polyacrylamide-gel, transferred in nitrocellulose membranes and immunoblotted with anti-NFRKB antibody.

### Electromobility Shift Assays

Cytosolic and nuclear fractions were prepared essentially as described previously [32]. Protein concentrations were assayed using the Bio-Rad dye reagent (Bio-Rad, Richmond, CA), following the instructions provided by the manufacturer. The double-stranded oligonucleotide probes (100 ng), with the consensus and mutant NF-kB (sc-2505, sc-2511), GAS/ISRE (sc-2537, sc-2538), NFATc (sc-2577, sc-2578) and AP1 (sc-2501, sc2514) sequences, respectively, were purchased from Santa Cruz Biotechnology (Santa Cruz, CA). The probes were labeled with [gamma-^32^P]ATP (3000 Ci/mmol) and purified on Chromaspin 30 columns (Clontech, Palo Alto, CA). Binding assays were performed as described previously [32].

### Genomic DNA preparation and methylated DNA quantification

HEK cells (1.5 10^6^) were seeded into 100 mm plates and 24 hours later, the transfection was performed with 8 microg by plate of pDest40 plasmid expressing NFRKB, or pDest40 empty vector, using nanofectine (PAA) according to the supplier's instructions. The transfected cells were incubated at 37°C under 5% CO2 for 24 h, then washed twice in 1× PBS, resuspended in 500 microl of buffer (50 mM Tris-HCl, 10 mM EDTA, 100 microg/ml DNase free RNaseA, pH8.0) and incubated for 30 min at 37°C. After RNAse treatment, the samples were incubated with 100 microl of Proteinase K (20 mg/ml) for 8 hours at 37°C. The DNA samples were extracted twice with phenol equilibrated in Tris–HCl pH 8 and once with Chloroform/Isoamyl alcool (24∶1). The DNA was ethanol-precipitated and the pellet dissolved in 500 microl sterile water. The concentration of the DNA samples was measured using a NanoDrop spectrophotometer. The global methylation level of the DNA samples was measured using the Inprint™ Methylated DNA quantification Kit (Sigma, Missouri, USA), according to the supplier's protocol. The percentage of methylated DNA was measured relatively to control DNA provided in the kit according the supplier's formula: [(sample minus blank)/(control minus blank)]×100.

### Statistical Analysis

Statistical analysis of the data was performed using PRIZM 4 for Macintosh (Graphpad Software, Inc, USA). Unpaired or Paired Student t-tests were used. P value of less than 0.05 was considered significant.
